# Essay on Cruelty to Animals

**Published:** 1839-10

**Authors:** 


ART. VII.—1. *Essay on Cruelty to Animals.* By JAMES MACAULAY, M.A. —*Edinburgh*, 1839. 8vo, pp. 135.

2. *The Animals' Friend; or, the Progress of Humanity.* Published periodically, for the Animals' Friend Society. No. VII.—*London*, 1839. 8vo, pp. 72.

WE are induced to call the attention of our readers to Mr. Macaulay's unpretending volume, from the fact that, although addressed to the public at large, it presents many subjects for serious reflection to the professional student. The author was formerly in the medical profession; and the calmness and ability with which he states his views show that he was properly qualified for the task. To this essay the Theological Faculty of Edinburgh have already awarded a prize; and we believe that much good will be done by its diffusion. It would be foreign to our purpose to give an abstract of this work. The whole subject is treated in a very systematic manner, and the conclusions are well supported by sound reasoning.

A considerable portion of the volume is devoted to an analysis of the supposed benefits which have been conferred on science by experiments on living animals. While Mr. Macaulay treats this part of the subject without prejudice, we think he bestows no more than a just censure upon many physiologists who have experimented on animals, not so much to add to the facts of science as to support their own peculiar doctrines. Magendie's experiments in France, and those of Dr. W. Phillip in this country, have very much this character. The one comes to the conclusion that the nerves are nothing more than electric conductors, and that the stomach digests food by a galvanic power: the other, that the stomach is of no use in vomiting, since, when it was cut away, and a pig's bladder substituted, there was no difference in the effects! It would be a curious point for enquiry, as to how many animals these two physiologists have destroyed, to acquire opinions which are now only retained by themselves. In a preceding article in the present Number, we have shown the inconclusiveness of many of Magendie's experiments on living animals, in relation to the properties of the blood. The author, who is entitled to speak from his personal acquaintance with that physiologist, fully confirms the opinion which we have expressed.

"M. Magendie himself, whose name and reputation are so identified with our subject, is not more successful than any of his colleagues in his hospital practice, notwithstanding all the light which he conceives he throws upon the nature of disease by his dissections. His mind is, indeed, counteracted by prejudices, derived from his peculiar pursuits, so that in a coarse and illiberal manner, he affects to despise and ridicule the researches of pathologists; and he is constantly falling into errors from rash generalizations, founded upon observations of the animal economy in unnatural states." (p. 91.)

We agree with the author, that the severest experiments are justifiable, when they are undertaken with a view to ascertain any definite and important point from which improvement in practice may reasonably be expected to result, but under no other circumstances whatever. We commend this volume to those of our readers who take an interest in the subject.

The second publication on our list speaks for itself. Its object and design are most praiseworthy, and we doubt not that it has already done much good. We are happy to make known to our readers the benevolent society from which it emanates. It would be strange if the professors of the medical art, whose special object is to relieve the pains of man, should be insensible to the sufferings of his inferior brethren.

